# Occipital Artery Function during the Development of 2-Kidney, 1-Clip Hypertension in Rats

**DOI:** 10.1155/2014/659617

**Published:** 2014-07-22

**Authors:** Stephen P. Chelko, Chad W. Schmiedt, Tristan H. Lewis, Tom P. Robertson, Stephen J. Lewis

**Affiliations:** ^1^Department of Cardiology, School of Medicine, Johns Hopkins University, Baltimore, MD 21205, USA; ^2^Department of Small Animal Medicine and Surgery, College of Veterinary Medicine, The University of Georgia, Athens, GA 30602, USA; ^3^Department of Physiology and Pharmacology, College of Veterinary Medicine, The University of Georgia, Athens, GA 30602, USA; ^4^Department of Pediatrics, School of Medicine, Case Western Reserve University, Cleveland, OH 44106-4984, USA

## Abstract

This study compared the contractile responses elicited by angiotensin II (AII), arginine vasopressin (AVP), and 5-hydroxytryptamine (5-HT) in isolated occipital arteries (OAs) from sham-operated (SHAM) and 2-kidney, 1-clip (2K-1C) hypertensive rats. OAs were isolated and bisected into proximal segments (closer to the common carotid artery) and distal segments (closer to the nodose ganglion) and mounted separately on myographs. On day 9, 2K-1C rats had higher mean arterial blood pressures, heart rates, and plasma renin concentrations than SHAM rats. The contractile responses to AII were markedly diminished in both proximal and distal segments of OAs from 2K-1C rats as compared to those from SHAM rats. The responses elicited by AVP were substantially greater in distal than in proximal segments of OAs from SHAM rats and that AVP elicited similar responses in OA segments from 2K-1C rats. The responses elicited by 5-HT were similar in proximal and distal segments from SHAM and 2K-1C rats. These results demonstrate that continued exposure to circulating AII and AVP in 2K-1C rats reduces the contractile efficacy of AII but not AVP or 5-HT. The diminished responsiveness to AII may alter the physiological status of OAs *in vivo*.

## 1. Introduction

The cell bodies of vagal afferents are located in the nodose ganglia [1, 2]. Since vagal afferents are true bipolar cells, alterations in resting potential of the cell bodies will influence transmission of centrally directed action potentials to the nucleus tractus solitarius [1, 2]. Jacobs and Comroe [3] demonstrated that injections of 5-hydroxytryptamine (5-HT) or the selective 5-HT_3_ receptor agonist, phenyldiguanide, into the common carotid artery (CCA) of cats elicited rapid falls in arterial blood pressure and heart rate which were prevented by ligation of the occipital arteries (OAs). In more recent studies, Lacolley et al. [1, 2] provided evidence that the OA blood supply to the nodose ganglia of rats lacks an effective blood-ganglion barrier and that circulating 5-HT gains access to and activates nodose ganglion cell bodies exclusively via the OAs. These findings raise the possibility that blood-borne factors in the OA microcirculation may regulate vagal afferent transmission via actions on receptors, ion channels, and enzymes on plasma membranes of nodose ganglion cell bodies (see [1, 2, 4–8]), including angiotensin II (AII) receptors (7), arginine vasopressin (AVP) receptors [8], and 5-HT receptors (see [1, 2]). In turn, the functional status of the OA microvasculature would play a pivotal role in allowing these circulating factors to gain access to vagal afferent cell bodies (see [1, 2]). As such, agents that constrict or dilate the OA or downstream microvessels in the nodose ganglia would regulate the delivery of blood-borne factors to vagal afferent cell bodies (see [1, 2, 9]).

The likelihood that OAs respond to circulating factors is supported by evidence that 5-HT contracts OAs harvested from anesthetized patients with brain tumors and/or aneurysms via activation of 5-HT_1B_ and 5-HT_2A_ receptors [10]. Moreover, OAs taken from Sprague-Dawley rats constrict upon activation of 5-HT_1A_ and 5-HT_2_ receptors, angiotensin AT_1_ receptors, and AVP_1_ receptors [9]. Renovascular hypertension is the most common cause of secondary hypertension [11, 12]. The hypertension resulting from a decrease in renal blood flow is initiated via activation of the renin-angiotensin-aldosterone system [13–15]. Renovascular hypertension is associated with increases in circulating vasoconstrictors such as AII and AVP [15, 16], which decrease arteriolar diameter, thereby raising vascular resistance and arterial blood pressure [17]. In addition, AII and AVP influence renal absorption of sodium and water via aldosterone release, thereby increasing blood volume and cardiac output [18]. Taken together with the knowledge that blood-borne factors gain ready access to nodose ganglion cell bodies, it is possible that circulating AII and AVP may contribute to the etiology of renovascular hypertension via actions on vagal afferent cell bodies. However, the ability of these compounds to affect vagal afferent function will be regulated by their effects on OA blood flow to the nodose ganglion.

To date, no studies have examined the contractile properties of OAs upon development of 2-kidney, 1-clip (2K-1C) induced hypertension, an established animal model of human renovascular hypertension [19–22]. As such, this study compared the responses elicited by AII, AVP, and 5-HT in OAs isolated from sham-operated rats and those with 2K-1C hypertension. This study revealed the novel finding that the vasoconstrictor responses elicited by AII but not AVP or 5-HT were markedly reduced in OAs from 2K-1C rats.

## 2. Materials and Methods

### 2.1. Rats

This investigation conforms to the Guide for the Care and Use of Laboratory Animals from the National Institutes of Health (NIH Publication number 85-23, revised 1996). All protocols were approved by the University of Georgia Animal Care and Use Committee.

### 2.2. Experimental Groups and Surgeries

Male Sprague-Dawley rats (380–400 g) were assigned to two experimental study groups (*n* = 8 per group). All rats were weighed and anesthetized via 2% isoflurane delivered in 95% O_2_ and 5% CO_2_. The left kidney was exteriorized via a left paracostal celiotomy. The renal artery and vein were carefully isolated by blunt dissection. A titanium clip was placed on the left renal artery and a nylon suture (5-0 Ethilon, Ethicon, Inc., Somerville, NJ) was tied through a predrilled hole to prevent clip dislodgement, as described previously [23]. The renal clips were manufactured from medical grade titanium (0.25 mm internal gap width). The efficacy of eliciting renovascular hypertension in rats using these clips has been described previously [23]. Sham surgeries were performed as above except that the clip was placed on the renal artery for 15–20 sec and then quickly removed. The abdominal wall and skin were sutured (5-0 Ethilon, Ethicon, Inc., Somerville, NJ) and stapled (9 mm staples, Reflex 9, Cellpoint Scientific, Inc., Gaithersburg, MD), respectively.

### 2.3. Blood Pressure Measurements and Plasma Renin Concentration

Nine days after sham- or renal-clipping, the sham-operated (SHAM) and 2K-1C rats were weighed and anesthetized with 2% isoflurane delivered in 95% O_2_ and 5% CO_2_. A polyethylene catheter (PE-3, Scientific Commodities Inc., Lake Havasu City, AZ) was advanced inside the left femoral artery. Mean and peak systolic arterial blood pressures were measured via a pressure transducer (Radnoti LLC, Monrovia, CA) connected to a data acquisition system (LabChart, AD Instruments, Colorado Springs, CO). Blood was collected in EDTA (7.2 mg, Vacutainer, BD, Franklin Lakes, NJ) via the arterial catheter. Plasma renin concentrations were measured in triplicate via a fluorometric kit (SensoLyte 520 Rat Renin Assay Kit, AnaSpec, Inc., Fremont, CA), as described previously [23, 24].

### 2.4. Tissue Weight Parameters

Following blood collection, the rats were killed by decapitation under isoflurane anesthesia. Heart weights and the weights of both kidneys were recorded. In addition, heart weight/body weight and kidney weight/body weight ratios were also determined.

### 2.5. Artery Isolation and Small Vessel Myography

Following euthanasia, the rat heads were immediately placed in ice cold physiological saline solution (PSS) containing (mM) NaCl 118, NaHCO_3_ 24, KCl 4, glucose 5.6, MgSO_4_ 1, NaH_2_PO_4_ 0.435, and CaCl_2_ 1.8. OAs (250–400 *μ*m internal diameter) were isolated and bisected into proximal and distal segments (relative to the external carotid) and mounted separately on small vessel myographs (Model 500A, Danish Myo Technology, Denmark), as described previously [9]. After equilibrating for 30 min in PSS gassed with 12% O_2_, 5% CO_2_, and 83% N_2_ (pH 7.4, 37°C), OAs were stretched as described for systemic arteries [25]. Maximal contractile responses of OA segments to a depolarizing stimulus were established by exposing them to 80 mM K^+^ (KPSS; isotonic replacement of Na^+^ by K^+^; 3 × 2 min exposures, 15–20 min apart), as described previously [9, 26]. Concentration-dependent response curves were established for AII (100 pM to 100 nM), AVP (100 pM to 100 nM), and 5-HT (1 nM to 10 *μ*M) on OAs from sham-operated and renal-clipped rats mentioned before. To provide comparisons to the data derived from OAs, CCAs were isolated from the previously mentioned sham-operated and renal-clipped rats to determine the effects of AII, AVP, and 5-HT (as above).

### 2.6. Drugs

[Arg^8^]-Vasopressin acetate salt and 5-hydroxytryptamine hydrochloride were from Sigma-Aldrich (St. Louis, MO). Angiotensin II was from American Peptide Co. (Sunnyvale, CA).

### 2.7. Data and Statistical Analysis

All data are presented as mean ± SEM. Contractile responses elicited by the vasoconstrictors were expressed as a percentage of the maximal contractile response elicited by KPSS (*E*_max⁡_, %*T*_*K*_). Vasoconstrictor potency (EC_50_) values were calculated by nonlinear regression analyses (GraphPad Software, La Jolla, CA). All data were analyzed by one-way ANOVA or two-way repeated measures ANOVA using BMDP statistical software (Statistical Solutions, Boston, MA) followed by Bonferroni corrections for multiple comparisons between means using the error mean square term from the ANOVAs [27]. The *P* < 0.05 value was corrected for the number of comparisons to determine significance.

## 3. Results

### 3.1. Parameters in SHAM and 2K-1C Rats

Data relevant to the SHAM and 2K-1C rats as measured 9 days after surgery are summarized in Table 1. The 2K-1C rats had higher mean and peak systolic arterial blood pressures, heart rates, and plasma renin concentrations than the SHAM rats. The body weights of the two groups were similar to one another. It should be noted that the 2K-1C rats did not put on body weight over the 9 days of clipping (day 0 versus day 9; 415 ± 18 g on day 0 versus 413 ± 14 g on day 9; difference = −2 ± 6 g), whereas the SHAM rats did (417 ± 16 g on day 0 versus 426 ± 13 g on day 9; difference = +9 ± 4 g). The weights of nonclipped kidneys (actual weights and those corrected for body weight) were slightly higher in 2K-1C rats than in SHAM rats. The weights of clipped kidneys in 2K-1C rats were lower than those of the corresponding kidneys of SHAM rats. The heart weights of 2K-1C rats were greater than those of SHAM rats.

### 3.2. Contractile Responses in Proximal and Distal OA Segments

**Figure 1:**
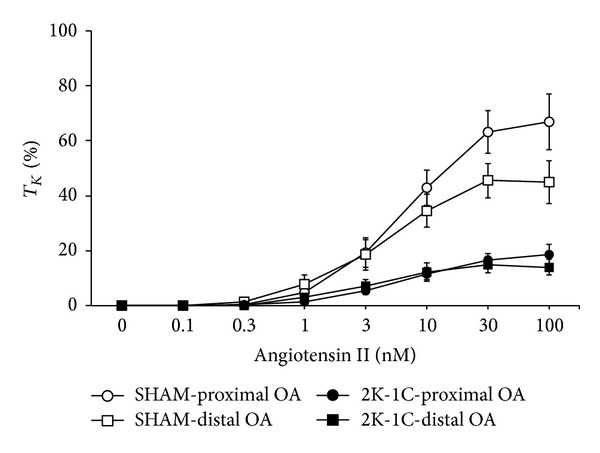
Dose-response effects of angiotensin II on tension of proximal and distal occipital artery segments from sham-operated (SHAM) and 2-kidney, 1-clip (2K-1C) hypertensive rats. The data are presented as mean ± SEM of the responses expressed as a percentage of that elicited by 80 mM K^+^ (%*T*_*K*_). There were 8 segments in each group.

The contractile responses elicited by AII in proximal and distal OA segments from SHAM and 2K-1C rats are summarized in Figure 1. AII elicited similar concentration-dependent contractions in proximal OA segments (i.e., those further from the nodose ganglion) and distal OA segments (i.e., those closer to the nodose ganglion) of SHAM rats. AII elicited similar contractions in proximal and distal segments from 2K-1C rats. The contractions elicited by AII in the proximal and distal segments from 2K-1C rats were smaller than those from SHAM rats. As summarized in Table 2, the concentrations eliciting half-maximal (EC_50_) responses in proximal and distal segments were similar to one another in SHAM and 2K-1C arteries. The EC_50_ values for AII in segments from 2K-1C rats were similar to those of corresponding segments from SHAM rats. The maximal responses (*E*_max⁡_, %*T*_*K*_) elicited by AII were similar in proximal and distal OA segments from SHAM rats and from 2K-1C rats (Table 2). However, the maximal responses elicited by AII in proximal and distal segments from 2K-1C rats were smaller than in those from SHAM rats.

**Figure 2:**
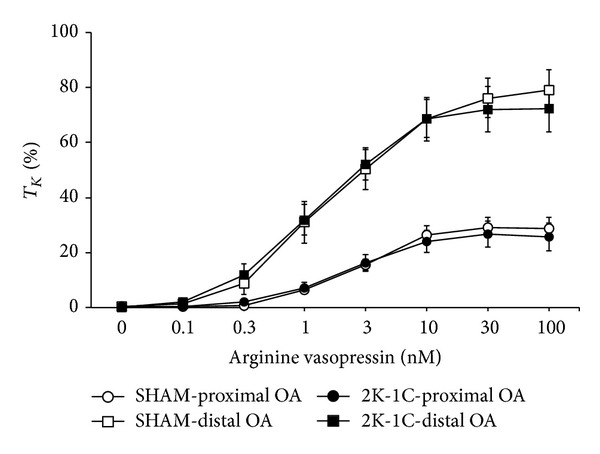
Dose-response effects of arginine vasopressin on tension of proximal and distal occipital artery segments from sham-operated (SHAM) and 2-kidney, 1-clip (2K-1C) hypertensive rats. The data are presented as mean ± SEM of the responses expressed as a percentage of that elicited by 80 mM K^+^ (%*T*_*K*_). There were 8 segments in each group.

AVP elicited concentration-dependent contractions in proximal and distal OA segments taken from SHAM and 2K-1C rats (Figure 2). AVP responses were greater in distal segments than in proximal segments from SHAM and 2K-1C rats. The contractions elicited by AVP in proximal and distal segments from 2K-1C rats were similar to those in the corresponding segments from SHAM rats. EC_50_ values were similar between proximal and distal OA segments from SHAM rats and from 2K-1C rats (Table 2). EC_50_ values were similar between SHAM and 2K-1C segments. The maximal responses (*E*_max⁡_, %*T*_*K*_) elicited by AVP in distal segments were greater than those in proximal segments from SHAM and 2K-1C rats (Table 2). The responses in proximal and distal segments from 2K-1C rats were similar to those in the respective segments from SHAM rats.

**Figure 3:**
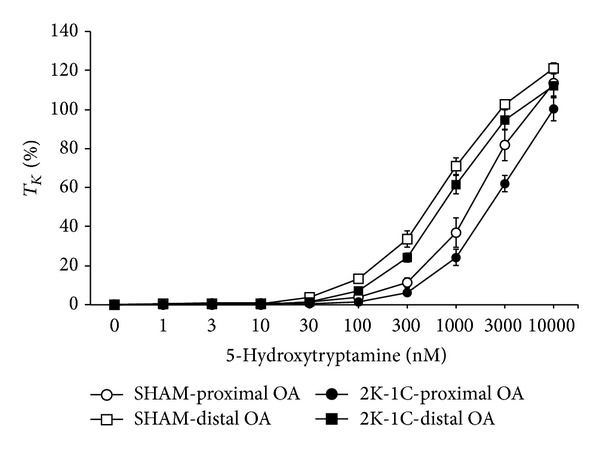
Dose-response effects of 5-hydroxytryptamine on tension of proximal and distal occipital artery segments from sham-operated (SHAM) and 2-kidney, 1-clip (2K-1C) hypertensive rats. The data are presented as mean ± SEM of the responses expressed as a percentage of that elicited by 80 mM K^+^ (%*T*_*K*_). There were 8 segments in each group.

5-HT elicited concentration-dependent contractions in OA segments from SHAM and 2K-1C rats (Figure 3). AVP responses were somewhat greater in distal OA segments (those closer to the nodose ganglion) than in proximal OA segments from SHAM and 2K-1C rats. 5-HT responses in proximal and distal segments from 2K-1C rats were similar to those in the same segments from SHAM rats. As seen in Table 2, the EC_50_ values in proximal segments were higher than in distal segments from SHAM and from 2K-1C rats (i.e., 5-HT was a more potent vasoconstrictor in distal segments). EC_50_ values were higher in 2K-1C proximal segments from 2K-1C rats than from SHAM rats (i.e., diminished potency in 2K-1C segments), whereas EC_50_ values were similar in distal segments from SHAM and 2K-1C rats. Maximal responses (*E*_max⁡_, %*T*_*K*_) elicited by 5-HT were similar in proximal and distal segments from SHAM and 2K-1C rats. The responses in the proximal and distal segments from 2K-1C rats were similar to those in the respective segments from SHAM rats.

### 3.3. Contractile Responses in CCAs

**Figure 4:**
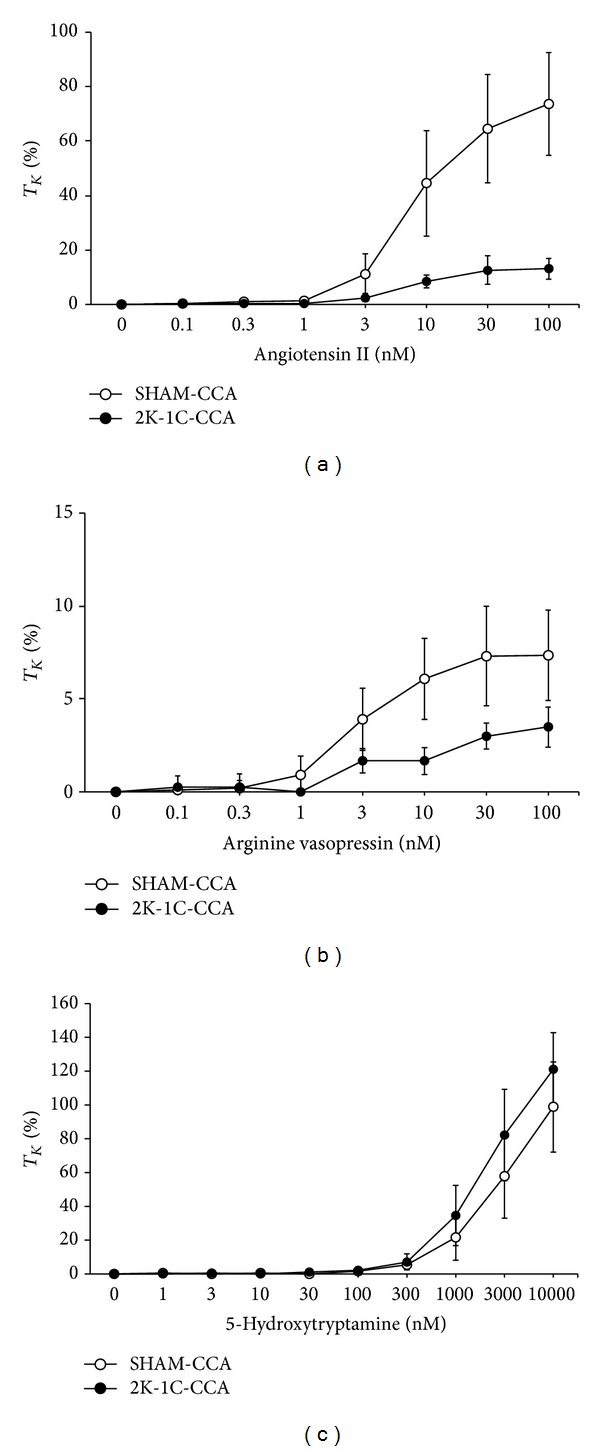
Dose-response effects of angiotensin II (a), arginine vasopressin (b), and 5-hydroxytryptamine (c) on tension of common carotid arteries from sham-operated (SHAM) and 2-kidney, 1-clip (2K-1C) hypertensive rats. The data are presented as mean ± SEM of the responses expressed as a percentage of that elicited by 80 mM K^+^ (%*T*_*K*_). There were 6 arteries in each group.

AII and 5-HT elicited robust responses in CCAs from SHAM rats whereas AVP elicited minor effects (Figure 4). The AII responses were smaller in CCAs from 2K-1C rats than from SHAM rats whereas the AVP and 5-HT responses were similar to those in CCAs of SHAM rats. EC_50_ values for AII in CCAs from SHAM and 2K-1C rats were similar to one another, whereas *E*_max⁡_ was smaller in CCAs from 2K-1C rats (Table 3). EC_50_ values for AVP were higher, whereas EC_50_ values for 5-HT were lower in CCAs from 2K-1C rats than from SHAM rats. *E*_max⁡_ values for AVP and 5-HT were similar in CCAs from 2K-1C and SHAM rats.

### 3.4. Comparison of Responses in CCAs and OAs

With respect to arteries from SHAM rats, (1) EC_50_ and *E*_max⁡_ values for AII were similar in CCAs and OAs, (2) EC_50_ values for AVP were similar in CCAs and OAs, whereas *E*_max⁡_ values were smaller in CCAs, and (3) EC_50_ values for 5-HT were higher in CCAs than in OAs, whereas *E*_max⁡_ values for 5-HT were similar in all arteries. With respect to arteries from 2K-1C rats, (1) *E*_max⁡_ values for AII were equally suppressed in CCAs and in OA segments compared to the corresponding arteries from SHAM rats, whereas there were no differences in EC_50_ values between arteries of SHAM and 2K-1C rats, (2) *E*_max⁡_ values for AVP were similar in all arteries from SHAM and 2K-1C rats, whereas EC_50_ values were higher in CCAs from 2K-1C rats than from SHAM rats (values similar in OA segments from SHAM and 2K-1C rats), and (3) *E*_max⁡_ values for 5-HT were similar in all arteries from SHAM and 2K-1C rats, whereas EC_50_ values were decreased in CCAs and increased in proximal OA segments but not different in distal segments from 2K-1C rats compared to those from SHAM rats.

## 4. Discussion

This study employed our novel renal artery clips to elicit 2K-1C hypertension in rats [23] and chose 9 days after clipping because each rat displays indices indicative of renovascular hypertension at this time (see [23]). As expected, the 2K-1C rats in the present study had elevated arterial blood pressures, heart rates, and plasma renin levels [19, 23, 28–30]. We did not measure circulating levels of AII or AVP but expected that these levels were elevated in our 2K-1C rats [19, 21, 22, 28–30]. Upon gross examination, the kidneys of both groups did not show signs of ischemic damage, consistent with our previous findings [23]. The findings that (1) the weights of the clipped kidneys in 2K-1C rats were less than the corresponding kidneys of SHAM rats, (2) the weights of the contralateral unclipped kidneys in 2K-1C rats were greater than the corresponding kidneys in SHAM rats, and (3) the hearts of 2K-1C rats were heavier (hypertrophied) than those of SHAM rats are hallmarks of this model [31–33]. Our finding that the 2K-1C rats had impaired weight gain is consistent with previous findings [23, 34]. Poor weight gain also occurs in humans with unilateral renal arterial stenosis, high renin concentrations, and increased blood pressure [35].

A major finding of this study was that AII elicited markedly smaller increases in tension in CCAs and proximal and distal OA segments from 2K-1C rats than from SHAM rats. We have reported that AII and the selective AT_1_ receptor agonist, val^5^-angiotensin II, contracted proximal and distal segments of OAs taken from conscious Sprague-Dawley rats, whereas no evidence was found for the presence of AT_2_ receptors in these OA segments [9]. As such, the diminished responses to AII in arteries from 2K-1C rats are probably due to the downregulation of AT_1_ receptors. The 2K-1C rats had elevated plasma renin levels at the time the arteries were removed and therefore plasma levels of AII were also likely elevated [19, 21, 22, 28–30]. As such, despite removing the arteries from the 2K-1C rats (and thereby exposure to circulating AII), placing them on wire myographs, and then exposing them to K^+^ to establish maximal tension development, the contractile responses to AII were still markedly diminished compared to arteries from SHAM rats. As will be discussed, the contractile responses to AVP and 5-HT were not diminished in OAs or CCAs from 2K-1C rats. Taken together, the diminished responses to AII in arteries from 2K-1C rats may have resulted from reduced AT_1_ receptor function on vascular smooth muscle during exposure to blood-borne AII* in vivo*, rather than to a disturbance of intracellular signaling pathways. Indeed, there is substantial evidence that AII elicits desensitization and downregulation (internalization) of AT_1_ receptors via multiple (e.g., *β*-arrestin-dependent) mechanisms [36–38].

The AII-mediated contractions in the OA segments were greater than those observed in our earlier study [9]. In the present study, arteries were dissected from rats anesthetized with isoflurane at decapitation, whereas, in our previous study, the rats were decapitated without anesthesia. Although this may suggest that any residual isoflurane in arteries may augment AII contractility, it is known that the direct application of isoflurane to organ baths holding rat aortae suppresses the contractile effects of AII [39–41]. The age and weights of the SHAM rats in the present study (16–18 weeks, 426 ± 13 g) were higher than the naïve rats (11-12 weeks, 350–380 g) used in our previous study [9]. This raises the possibility that the sensitivity of OAs to AII is age related (i.e., increases as the rats get older). This may be a unique phenomenon to OAs as age-dependent change in responsiveness to AII does not occur in a wide variety of arteries, including aorta and coronary, basilar, femoral, and mesenteric arteries [42–47].

AVP elicited robust increases in tension in distal OA segments from SHAM rats and substantially smaller contractions in proximal OA segments from these rats. AVP elicited even smaller responses in CCAs than in proximal OAs. The relative potencies of AVP in the proximal and distal OA segments were very similar to those reported in our earlier study [9]. Our previous study [9] demonstrated that the contractile effects of AVP in proximal and distal segments of OAs taken from conscious Sprague-Dawley rats were due to activation of V_1_ receptors and no evidence was found for the presence of vasorelaxant or vasoconstrictor AVP V_2_ receptors in these OA segments [9]. As such, the mechanisms that may have been responsible for the enhanced potency of AII in the present study (see above) do not obviously pertain to AVP V_1_ receptor signaling. Moreover, evidence that direct application of isoflurane to vessel baths holding rat aortae diminished the vasoconstrictor potency of AVP [48] also suggests that isoflurane did not have lingering effects on vascular contractility of OA segments to AVP (and perhaps AII) in the present study.

An important observation of the present study was that the contractile effects of AVP in both proximal and distal OA segments from 2K-1C rats were virtually identical to those of the corresponding segments from SHAM rats. It therefore appears that the mechanisms that elicit desensitization and/or downregulation of AII AT_1_ receptors in 2K-1C hypertensive rats do not affect AVP V_1_ receptors. However, it should be noted that the direct contractile effects of AVP are subject to tachyphylaxis in rat aortic rings [49]. Moreover, AVP V_1_ receptors are subject to both homologous and heterologous desensitization [50, 51] and downregulation/internalization [52] upon agonist activation by mechanisms involving activation of protein kinase C. It therefore appears that the elevated levels of circulating AVP found after clipping of one renal artery [19, 21, 22, 28–30] are not sufficient to modulate AVP V_1_ receptor signaling* in vivo* in our 2K-1C rats or, if present, this is lost by the time the contractile effects of AVP are tested* in vitro*.

5-HT and the selective 5-HT_2_ receptor agonist, *α*-methyl-5-HT, contract proximal and distal segments of OAs from conscious Sprague-Dawley rats that are abolished by the 5-HT_2_ receptor antagonist, ketanserin [9]. In the present study, 5-HT increased tension in proximal and distal OA segments and in CCAs of SHAM rats. The relative potencies of 5-HT in the proximal and distal OA segments were similar to those reported in our earlier study except that the more pronounced contractions in distal segments from the nonanesthetized rats occurred at 1 and 3 *μ*M [9]. It therefore appears that there is a subtle potentiating effect of isoflurane anesthesia (and/or age, see discussion above) on the contractile effects of 5-HT. However, evidence that direct application of isoflurane diminished the vasoconstrictor potency of 5-HT [53, 54] argues against the possibility that isoflurane had lingering effects on vascular contractility of OA segments to 5-HT in the present study. As with AVP, it appears that the mechanisms responsible for the enhanced potency of AII in the present study (see above) do not obviously pertain to 5-HT_2_ receptor signaling.

An important observation of the present study was that the contractile effects of 5-HT in CCAs and proximal and distal segments of OAs from 2K-1C rats were similar to those of the corresponding segments from SHAM rats. As such, the mechanisms that elicit desensitization and/or downregulation of AII AT_1_ receptors in 2K-1C hypertensive rats do not affect 5-HT_2_ receptors. It should be noted that the direct contractile effects of 5-HT on arteries are subject to tachyphylaxis via mechanisms in the smooth muscle [55, 56]. Moreover, 5-HT_2_ receptors are subject to desensitization and downregulation/internalization upon agonist activation [57]. It therefore appears that the elevated plasma levels of AII and AVP in 2K-1C rats (no evidence for increased plasma levels of 5-HT) are not sufficient to modulate 5-HT_2_ receptor signaling* in vivo*, or, if it does occur, it is lost by the time the effects of 5-HT are tested* in vitro*. It should also be noted that 5-HT-induced constrictions of OAs harvested from anesthetized humans occurred via activation of 5-HT_1B_ and 5-HT_2A_ receptors [10].

In summary, the potency and maximal effects of AII were similar in the CCA and proximal and distal segments of the OA. In contrast, AVP elicited minor responses in the CCA, slightly more pronounced responses in the proximal OA (that closest to the CCA), and pronounced responses in the distal OA (that closest to the nodose ganglion). The graded increase in the maximum responses to AVP from the CCA to the distal OA in SHAM and in 2K-1C rats (see Tables 2 and 3) suggests that AVP receptors may play an important role in regulating the microcirculation within the nodose ganglion itself. Moreover, the increase in potency of 5-HT from the carotid artery to proximal OA to distal OA in SHAM and in 2K-1C rats (see Tables 2 and 3) also suggests that 5-HT receptors in the microcirculation of the nodose ganglion will have a meaningful role in regulating vascular tone in conditions associated with elevated circulating levels of 5-HT. Although we demonstrated that blood renin levels are elevated in the 2K-1C rats, we did not measure circulating AII, AVP, or 5-HT levels. As mentioned above, we would expect the blood levels of AII and AVP to be elevated [19, 21, 22, 28–30], whereas to our knowledge nothing is known as to the status of circulating 5-HT in 2K-1C rats. We are beginning to perform these blood measurements and important studies that will determine the densities and affinities of AII, AVP, and 5-HT receptor subtypes within the CCA and proximal and distal OA segments of SHAM and 2K-1C rats.

Our finding that the contractile effects of AII on OAs of 2K-1C rats were diminished whereas the contractile effects of AVP and 5-HT were not has direct implications for the status of blood flow to nodose ganglia and the delivery of blood-borne factors to vagal afferent cells bodies within these ganglia* in vivo* (see [1, 2]). It is known that infusions of AII or lysine vasopressin actually increased the diameter of CCAs in rats [58]. It therefore appears that the increases in arterial blood pressure due to constriction of resistance arteries in systemic (e.g., mesenteric, hepatic, and femoral) beds outweigh the direct constrictor effects of the peptides on the CCAs. It could be expected that the situation would be similar in the OAs in which the arterial pressure effects of AII and AVP may dominate those of the direct constrictor effects on the OAs (which are not true resistance arteries), whereas the constrictor effects of the peptides in the microcirculation of the nodose ganglia would dominate. As such, the constrictor effects of the peptides would limit access of these and other circulating factors to the afferent cell bodies within the nodose ganglia. The downregulation of angiotensin AT_1_ receptors on smooth muscle of OAs and potentially the downstream resistance arterioles within the nodose ganglia during the progression of 2K-1C hypertension would thereby affect the overall contractile status of the OA vascular network and therefore the access of circulating factors to nodose ganglion cells. Whether the loss of AII vasoconstriction has a functional impact on OA blood flow to the nodose ganglia in face of the continued presence (and efficacy) of circulating AVP in 2K-1C rats awaits investigation.

AII [7] and AVP [8] directly affect the activity of nodose ganglion cells* in vitro*. As such, the access of blood-borne AII and AVP to nodose ganglion cells in 2K-1C animals would have the capacity to modulate vagal afferent activity and therefore the expression of hypertension and disturbances in baroreceptor reflex function in these animals (see [1, 2, 21, 22]) and perhaps affect the ability of nodose ganglion cells to synthesize AII and AVP receptors designated for deposition onto the cell membranes of the afferent cell bodies and for axonal transport to central and/or peripheral terminals (see [7, 8, 59]).

## Figures and Tables

**Table 1:** Body and tissue weight parameters in SHAM and 2K-1C rats on day 9.

Parameter	SHAM	2K-1C
Mean arterial blood pressure, mmHg	96 ± 1	120 ± 4∗
Peak systolic arterial blood pressure, mmHg	105 ± 3	134 ± 5∗
Heart rate, beats/min	340 ± 12	396 ± 9∗
Plasma renin concentration, *μ*g/mL	68 ± 2	106 ± 13∗
Body weight, g	426 ± 13	413 ± 14
Nonclipped kidney weight, g	1.2 ± 0.04	1.4 ± 0.06∗
Nonclipped kidney weight/body weight, mg/g	2.9 ± 0.08	3.3 ± 0.09∗
Clipped kidney weight, g	1.2 ± 0.05	1.0 ± 0.03∗
Clipped kidney weight/body weight, mg/g	2.8 ± 0.08	2.4 ± 0.09∗
Heart weight, g	1.35 ± 0.04	1.48 ± 0.06∗
Heart weight/body weight, mg/g	3.2 ± 0.04	3.6 ± 0.08∗

The data are presented as mean ± SEM. SHAM: sham-operated rats; 2K-1C: 2-kidney, 1-clip rats. There were 8 rats in each group. *P < 0.05 in 2K-1C versus SHAM.

**Table 2:** Contractile effects of agonists on proximal and distal segments of the occipital artery.

Agonist	Parameter	Proximal segments		Distal segments	
		SHAM	2K-1C	SHAM	2K-1C
Angiotensin II	EC_50_, nM	6.5 ± 1.3	6.8 ± 1.5	4.1 ± 1.4	3.0 ± 1.6
	*E*_max⁡_, %*T*_*K*_	66.8 ± 10.1	18.5 ± 3.8∗	44.9 ± 7.8	14.0 ± 2.9∗
AVP	EC_50_, nM	2.5 ± 1.3	2.2 ± 1.5	1.5 ± 1.5	1.2 ± 1.5
	*E*_max⁡_, %*T*_*K*_	28.8 ± 4.0	25.7 ± 5.0	79.2 ± 7.3^†^	72.3 ± 8.6^†^
5-HT	EC_50_, nM	1,941 ± 119	2,852 ± 120∗	844 ± 110^†^	941 ± 113^†^
	*E*_max⁡_, %*T*_*K*_	113.3 ± 6.5	100.3 ± 6.2	121.3 ± 2.8	112.2 ± 6.2

Data are presented as mean ± SEM. AVP: arginine vasopressin. 5-HT: 5-hydroxytryptamine. EC_50_: concentration causing 50% maximal contraction. *E*_max⁡_ %*T*_*K*_: maximal contraction as a percentage of the response elicited by 80 mM K^+^. There were 8 artery segments in each group. *P < 0.05 in 2K-1C versus SHAM; ^†^ *P* < 0.05 in distal versus proximal segments.

**Table 3:** Contractile effects of angiotensin II, AVP, and 5-HT receptor agonists in common carotid arteries from 2K-1C and SHAM rats.

Constrictor	EC_50_, nM		*E*_max⁡_, %*T*_*K*_	
	SHAM	2K-1C	SHAM	2K-1C
Angiotensin II	7.9 ± 1.2	7.0 ± 1.2	73.6 ± 18.9	13.1 ± 3.9∗
AVP	2.8 ± 1.5	10.2 ± 1.9∗	7.4 ± 3.5	3.5 ± 1.1
5-HT	3,246 ± 144	2,204 ± 121∗	98.8 ± 26.7	121.0 ± 21.4

Data are presented as mean ± SEM. AVP: arginine vasopressin. 5-HT: 5-hydroxytryptamine. EC_50_: concentration causing 50% maximal contraction. *E*_max⁡_, %*T*_*K*_: maximal contraction as a percentage of the response elicited by 80 mM K^+^. There were 6 artery segments in each group. *P < 0.05 in 2K-1C versus SHAM.

## References

[1] Lacolley P, Owen JR, Sandock K (2006). Occipital artery injections of 5-HT may directly activate the cell bodies of vagal and glossopharyngeal afferent cell bodies in the rat. *Neuroscience*.

[2] Lacolley P, Owen JR, Sandock K (2006). 5-HT activates vagal afferent cell bodies *in vivo*: role of 5-HT_2_ and 5-HT_3_ receptors. *Neuroscience*.

[3] Jacobs L, Comroe JH (1971). Reflex apnea, bradycardia, and hypotension produced by serotonin and phenyldiguanide acting on the nodose ganglia of the cat. *Circulation Research*.

[4] Lee CH, Sun SH, Lin SH, Chen CC (2011). Role of the acid-sensing ion channel 3 in blood volume control. *Circulation Journal*.

[5] Li Y, Zheng H (2011). Angiotensin II-NADPH oxidase-derived superoxide mediates diabetes-attenuated cell excitability of aortic baroreceptor neurons. *The American Journal of Physiology—Cell Physiology*.

[6] Zhao H, Kinch DC, Simasko SM (2011). Pharmacological investigations of the cellular transduction pathways used by cholecystokinin to activate nodose neurons. *Autonomic Neuroscience: Basic and Clinical*.

[7] Widdop RE, Krstew E, Jarrott B (1990). Temperature dependece of angiotensin II-mediated depolarisation of the rat isolated nodose ganglion. *European Journal of Pharmacology*.

[8] Gao X, Phillips PA, Widdop RE, Trinder D, Jarrott B, Johnston CI (1992). Presence of functional vasopressin V1 receptors in rat vagal afferent neurones. *Neuroscience Letters*.

[9] Chelko SP, Schmiedt CW, Lewis TH (2013). Vasopressin-induced constriction of the isolated rat occipital artery is segment dependent. *Journal of Vascular Research*.

[10] Verheggen R, Meier A, Werner I (2004). Functional 5-HT receptors in human occipital artery. *Naunyn-Schmiedeberg's Archives of Pharmacology*.

[11] Berglund G, Andersson O, Wilhelmsen L (1976). Prevalence of primary and secondary hypertension: studies in a random population sample. *British Medical Journal*.

[12] Strandness DE (1994). Natural history of renal artery stenosis. *American Journal of Kidney Diseases*.

[13] Goldblatt H, Lynch J, Hanzal RF, Summerville WW (1934). Studies on experimental hypertension: I. The production of persistent elevation of systolic blood pressure by means of renal ischemia. *The Journal of Experimental Medicine*.

[14] Goldblatt H, Haas E, Klick RL, Lewis L (1976). The effect of main artery occlusion of one kidney on blood pressure of dogs. *Proceedings of the National Academy of Sciences of the United States of America*.

[15] Safian RD, Textor SC (2001). Renal-artery stenosis. *The New England Journal of Medicine*.

[16] Dieter RS, Schmidt WS, Pacanowski JP, Jaff MR (2005). Renovascular hypertension. *Expert Review of Cardiovascular Therapy*.

[17] Kitazawa T, Eto M, Woodsome TP, Brautigan DL (2000). Agonists trigger G protein-mediated activation of the CPI-17 inhibitor phosphoprotein of myosin light chain phosphatase to enhance vascular smooth muscle contractility. *Journal of Biological Chemistry*.

[18] Brewster UC, Setaro JF, Perazella MA (2003). The renin-angiotensin-aldosterone system: cardiorenal effects and implications for renal and cardiovascular disease states. *American Journal of the Medical Sciences*.

[19] Morgan T (2003). Renin, angiotensin, sodium and organ damage. *Hypertension Research*.

[20] Koyama T, Hatanaka Y, Jin X (2010). Altered function of nitrergic nerves inhibiting sympathetic neurotransmission in mesenteric vascular beds of renovascular hypertensive rats. *Hypertension Research*.

[21] Kumagai H, Suzuki H, Ryuzaki M, Matsukawa S, Saruta T (1990). Baroreflex control of renal sympathetic nerve activity is potentiated at early phase of two-kidney, one-clip goldblatt hypertension in conscious rabbits. *Circulation Research*.

[22] Kumagai H, Suzuki H, Ichikawa M (1993). Central and peripheral vasopressin interact differently with sympathetic nervous system and renin-angiotensin system in renal hypertensive rabbits. *Circulation Research*.

[23] Chelko SP, Schmiedt CW, Lewis TH, Lewis SJ, Robertson TP (2012). A novel vascular clip design for the reliable induction of 2-kidney, 1-clip hypertension in the rat. *Journal of Applied Physiology*.

[24] Schmiedt CW, Hurley KAE, Tong X, Rakhmanova VA, Po CL, Hurley DJ (2009). Measurement of plasma renin concentration in cats by use of a fluorescence resonance energy transfer peptide substrate of renin. *American Journal of Veterinary Research*.

[25] Mulvany MJ, Halpern W (1977). Contractile properties of small arterial resistance vessels in spontaneously hypertensive and normotensive rats. *Circulation Research*.

[26] Robertson TP, Ward JPT, Aaronson PI (2001). Hypoxia induces the release of a pulmonary-selective, Ca^2+^-sensitising, vasoconstrictor from the perfused rat lung. *Cardiovascular Research*.

[27] Wallenstein S, Zucker CL, Fleiss JL (1980). Some statistical methods useful in circulation research. *Circulation Research*.

[28] Katholi RE (1983). Renal nerves in the pathogenesis of hypertension in experimental animals and humans. *The American Journal of Physiology*.

[29] Barres CP, Lewis SJ, Grosskreutz CL, Varner KJ, Brody MJ (1989). Role of renal nerves in experimental hypertension: Evaluation of neurogenic mechanisms. *Clinical and Experimental Hypertension A*.

[30] Martinez-Maldonado M (1991). Pathophysiology of renovascular hypertension. *Hypertension*.

[31] Doggrell SA, Brown L (1998). Rat models of hypertension, cardiac hypertrophy and failure. *Cardiovascular Research*.

[32] Okuniewski R, Davis EA, Jarrott B, Widdop RE (1998). A comparison of the development of renal hypertension in male and female rats. *Clinical Science*.

[33] Helle F, Vågnes ØB, Iversen BM (2006). Angiotensin II-induced calcium signaling in the afferent arteriole from rats with two-kidney, one-clip hypertension. *American Journal of Physiology-Renal Physiology*.

[34] Muller DN, Klanke B, Feldt S (2008). (Pro)renin receptor peptide inhibitor “Handle-region” peptide does not affect hypertensive nephrosclerosis in Goldblatt rats. *Hypertension*.

[35] Uhari M, Heikkinen E (1981). Impaired weight gain and renovascular hypertension. *European Journal of Pediatrics*.

[36] Hunyady L, Catt KJ, Clark AJL, Gáborik Z (2000). Mechanisms and functions of AT_1_ angiotensin receptor internalization. *Regulatory Peptides*.

[37] Guo DF, Sun YL, Hamet P, Inagami T (2001). The angiotensin II type 1 receptor and receptor-associated proteins. *Cell Research*.

[38] Lymperopoulos A (2011). GRK2 and *β*-arrestins in cardiovascular disease: something old, something new. *American Journal of Cardiovascular Disease*.

[39] Yu J, Ogawa K, Tokinaga Y, Iwahashi S, Hatano Y (2004). The vascular relaxing effects of sevoflurane and isoflurane are more important in hypertensive than in normotensive rats. *Canadian Journal of Anesthesia*.

[40] Ishikawa A, Ogawa K, Tokinaga Y, Uematsu N, Mizumoto K, Hatano Y (2007). The mechanism behind the inhibitory effect of isoflurane on angiotensin II-induced vascular contraction is different from that of sevoflurane. *Anesthesia and Analgesia*.

[41] Qi F, Ogawa K, Tokinaga Y, Uematsu N, Minonishi T, Hatano Y (2009). Volatile anesthetics inhibit angiotensin II-induced vascular contraction by modulating myosin light chain phosphatase inhibiting protein, CPI-17 and regulatory subunit, MYPT1 phosphorylation. *Anesthesia and Analgesia*.

[42] Toda N, Hayashi S (1979). Age-dependent alteration in the response of isolated rabbit basilar arteries to vasoactive agents. *Journal of Pharmacology and Experimental Therapeutics*.

[43] Owen TL (1985). Reactivity of small vessels from mature to senescent female rabbits. *Blood Vessels*.

[44] Vanhoutte PM (1988). Aging and vascular responsiveness. *Journal of Cardiovascular Pharmacology*.

[45] Wakabayashi I, Sakamoto K, Hatake Yoshimoto KS, Kurahashi M (1990). Effect of age on contractile response to angiotensin II in rat aorta. *Life Sciences*.

[46] Tschudi MR, Luscher TF (1995). Age and hypertension differently affect coronary contractions to endothelin-1, serotonin, and angiotensins. *Circulation*.

[47] Konishi C, Naito Y, Saito Y, Ohara N, Ono H (1997). Age-related differences and roles of endothelial nitric oxide and prostanoids in angiotensin II responses of isolated, perfused mesenteric arteries and veins of rats. *European Journal of Pharmacology*.

[48] Shimogai M, Ogawa K, Tokinaga Y, Yamazaki A, Hatano Y (2010). The cellular mechanisms underlying the inhibitory effects of isoflurane and sevoflurane on arginine vasopressin-induced vasoconstriction. *Journal of Anesthesia*.

[49] Hamel C, Millette E, Lamontagne D (2005). Role of nitric oxide and protein kinase C in the tachyphylaxis to vasopressin in rat aortic rings. *Life Sciences*.

[50] Ancellin N, Preisser L, Le Maout S (1999). Homologous and heterologous phosphorylation of the vasopressin V1a receptor. *Cellular Signalling*.

[51] Ancellin N, More A (1998). Homologous and heterologous acute desensitization of vasopressin V1a receptor in *Xenopus* oocytes. *Cellular Signalling*.

[52] Preisser L, Ancellin N, Michaelis L, Creminon C, Morel A, Corman B (1999). Role of the carboxyl-terminal region, di-leucine motif and cysteine residues in signalling and internalization of vasopressin V_1a_ receptor. *FEBS Letters*.

[53] Witzeling TM, Sill JC, Hughes JM, Blaise GA, Nugent M, Rorie DK (1990). Isoflurane and halothane attenuate coronary artery constriction evoked by serotonin in isolated porcine vessels and in intact pigs. *Anesthesiology*.

[54] Xiaoping Z, List WF (1996). The inhibition of serotonin evoked bovine coronary artery contraction by halothane, isoflurane and sevoflurane is endothelium-independent. *European Journal of Anaesthesiology*.

[55] White RP, Robertson JT (1987). Pharmacodynamic evaluation of human cerebral arteries in the genesis of vasospasm. *Neurosurgery*.

[56] Reviriego J, Marín J (1989). Effects of 5-hydroxytryptamine on human isolated placental chorionic arteries and veins. *British Journal of Pharmacology*.

[57] Björk K, Sjögren B, Svenningsson P (2010). Regulation of serotonin receptor function in the nervous system by lipid rafts and adaptor proteins. *Experimental Cell Research*.

[58] Rutschmann B, Evequoz D, Aubert J, Brunner HR, Waeber B (1998). Vasopressin dilates the rat carotid artery by stimulating V1 receptors. *Journal of Cardiovascular Pharmacology*.

[59] Allen AM, Lewis SJ, Verberne AJM, Mendelsohn FAO (1988). Angiotensin receptors and the vagal system. *Clinical and Experimental Hypertension*.

